# MicroRNA-21 and the clinical outcomes of various carcinomas: a systematic review and meta-analysis

**DOI:** 10.1186/1471-2407-14-819

**Published:** 2014-11-07

**Authors:** Wenjia Wang, Jinhui Li, Wei Zhu, Chen Gao, RuiJingfang Jiang, Wenxue Li, Qiansheng Hu, Bo Zhang

**Affiliations:** Department of Preventive Medicine, School of Public Health, Sun Yat-sen University, Zhongshan II Road, Guangzhou, 510080 PR China; State Key Laboratory of Genetic Engineering, Centre for Genetic Epidemiology, School of Life Sciences, Fudan University, Shanghai, 200433 PR China; Department of Toxicology, Guangzhou Centre for Disease Control and Prevention, Guangzhou, 510440 PR China; Department of Public Health Sciences, Karolinska Institutet, Stockholm, 11668 Sweden

**Keywords:** miR-21, Cancer, Prognosis, Meta-analysis

## Abstract

**Background:**

MicroRNA-21 (miR-21) has been suggested to play a significant role in the prognosis of carcinoma. The recognition of novel biomarkers for the prediction of cancer outcomes is urgently required. However, the potential prognostic value of miR-21 in various types of human malignancy remains controversial. The present meta-analysis summarises and analyses the associations between miR-21 status and overall survival (OS) in a variety of tumours.

**Methods:**

Eligible published studies were identified by searching the PubMed and Chinese Biomedicine databases. The patients’ clinical characteristics and survival results were pooled, and a pooled hazard ratio (HR) with 95% confidence intervals (95% CI) was used to calculate the strength of this association. A random-effects model was adopted, and then, meta-regression and subgroup analyses were performed. In addition, an analysis of publication bias was also conducted.

**Results:**

Twenty-seven eligible articles (including 31 studies) were identified that included survival data for 3273 patients. The pooled HR suggested that high miR-21 was clearly related to worse overall survival (HR = 2.27, 95% CI: 1.81-2.86), with a heterogeneity measure index of I^2^ = 76.0%, *p* = 0.001, showing that miR-21 might be a considerable prognostic factor for poor survival in cancer patients.

**Conclusions:**

MiR-21 might be a potentially useful biomarker for predicting cancer prognosis in future clinical applications.

**Electronic supplementary material:**

The online version of this article (doi:10.1186/1471-2407-14-819) contains supplementary material, which is available to authorized users.

## Background

MicroRNAs (miRNAs) are a class of endogenous, small (approximately 22 nucleotides), non-coding, highly conserved and single-stranded RNAs that negatively regulate mRNA and protein expression by forming base-pairs with target mRNAs and sequentially induce translational repression and mRNA cleavage [1, 2]. More than 50% of miRNA genes are frequently located at fragile sites and genomic regions involved in multiple cancers, which suggests their potentially important and complex role in cancer [3]. Previous studies have showed that miRNAs are involved in regulating many urgent biological processes, such as cellular differentiation, proliferation, metabolism, cell-cycle control, development, apoptosis and tumour development [4, 5]. It has been reported that if the target gene of the miRNA is a tumour suppressor or oncogene, the aberrant expression of the miRNA will lead to disruptions in the miRNA-target genes and induce a disease status and even cancer development [6].

MiR-21 stands out as the most commonly dramatically up-regulated miRNA in both solid and haematological malignancies [7], and it is associated with clinicopathological factors in a considerable proportion of human malignancies [8–15]. In addition, extensive studies have implicated its integral role in tumour pathogenesis and during all other stages of carcinogenesis. Some studies have confirmed that miR-21 down-regulates four tumour suppressor genes: maspin, programmed cell death 4 (PDCD4), tropomyosin1 (TPM1) and phosphatase and tensin homolog (PTEN), which are all involved in tumourigenesis, cell cycle control, apoptosis and metastasis [16–20]. There is some evidence that indicates that the level of miR-21 expression is significantly associated with the prognosis of tumour patients, suggesting that it might serve as a prognostic marker for human malignancy [21].

Prognostic factors may identify subsets of patients with a worse prognosis and facilitate the selection of a more aggressive treatment strategy. The discovery of molecular biological prognostic factors would be helpful in a more accurate prediction of clinical outcome and may also reveal novel predictive factors and therapeutic targets [22]. However, the existing prognostic and predictive factors still need more proof, and they should be applied with caution when choosing the optimal adjuvant treatment. It is of great importance to balance the threshold of determining if patients need further treatment to avoid overtreatment or insufficient treatment. The prognostic role of miR-21 might potentially enhance the preoperative selection of low-risk patients who can be treated with resection alone, while directing high-risk cases to systemic treatment [23]. Above all, due to the apparent difference in expression between normal and malignant tissue and its causal role in cancer development, miR-21 is currently attracting considerable attention and has led to a number of studies reporting the relationship between miR-21 status and clinical outcomes among a wide variety of tumour types. However, most studies were conducted with a small sample size, and the observed associations were discordant. Therefore, we performed a literature-based meta-analysis of eligible studies to produce evidence-based results on the prognostic role of miR-21 in multiple types of malignant tumours to clarify this question and identify further research needs.

## Methods

We performed this meta-analysis according to the guidelines of the Meta-analysis of Observational Studies in Epidemiology group (MOOSE) [24] and PRISMA (Preferred Reporting Items for Systematic Reviews and Meta-analysis) [25].

### Search strategy and selection criteria

Studies were identified via an electronic search of PubMed and Chinese Biomedicine databases using the following keywords: (microRNA-21 OR miR-21 OR miR-21 OR mir21) AND (prognosis OR prognostic OR outcome OR mortality OR survival). The search ended on June 19^th^, 2014, and no lower date limit was used. The search was performed without language restriction. We also contacted some of the authors of the identified studies to obtain some unavailable data. Reference lists from relevant primary studies and review articles were also scanned for additional relevant publications. To ensure the quality of the meta-analysis, two authors (Li Jinhui & Wang Wenjia) independently performed the search and identification according to the standardised approach, and the final selection of a study for inclusion in the meta-analysis was reached by consensus.

To be eligible for inclusion, studies met the following criteria: (I) they reported research on patients with any type of carcinoma; (II) they measured the expression of miR-21 and reported the corresponding cut-off value; (III) they investigated the association between miR-21 expression and overall survival (OS); (IV) the hazard ratio (HR) for overall survival according to miR-21 status either had to be reported or could be calculated from the information presented; (V) the study sample size was higher than twenty individuals; (VI) when the same author or group reported results obtained from the same patient population in more than one article, the most recent report or the most informative one was included in this analysis to avoid overlapping between cohorts; and (VII) they used tissue samples (without any neoadjuvant therapy) obtained from surgically resected tumours and corresponding noncancerous or normal tissues for comparison.

### Definition, data extraction and methodological assessment

Overall survival was defined as the interval between the medical treatment and the death of patients or the last observation.

All eligible publications were reviewed by two reviewers (Li Jinhui & Wang Wenjia), and they then extracted the study data based on a predefined standardised form including the characteristics of eligible studies, the baseline information of patients and the survival analysis data (Additional file 1: Table S1). Disagreements were resolved by discussion. The extracted information was summarised in a consistent manner to prevent bias. Survival outcome data were synthesised using the time-to-event hazard ratio (HR) and the 95% confidence intervals (95% CI) from the original article as the effective measure. If this information was not available, sending an email to the authors for complementary information was our first choice. If the Kaplan-Meier survival curves were available, we used the method previously described by Parmar et al. and Tierney et al. to estimate HR and its corresponding 95% CI [26]. Additional data were extracted from the studies, including the first author, publication year, number of patients, mean age, follow-up, cancer type (system), cancer category and stage.

Furthermore, a methodological assessment of each study was also conducted by two investigators (Li Jinhui & Wang Wenjia) according to REMARK guidelines [27]. Disagreements were adjudicated by a third investigator (Zhu Wei) after referring to the original articles.

### Statistical analysis

To quantitatively combine the survival data, we extracted the HRs and their 95% CIs to assess the impact of the miR-21 status on tumour prognosis. A combined HR > 1 implied a worse survival for the group with miR-21 overexpression. This negative impact of miR-21 on survival was considered statistically significant if the 95% CI for the combined HR did not overlap 1. To assess heterogeneity among the studies, we used I^2^ statistics, which describe the proportion of total variation in meta-analysis estimates due to between-study heterogeneity. The variation is measured from 0-100%, with increasing I^2^ values indicating a larger impact of between-study heterogeneity in the meta-analysis [28]. The results were considered statistically significant if the *p* value was less than 0.05 and was quantified using the I^2^ metric (I^2^ < 25%, no heterogeneity; I^2^ = 25-50%, moderate heterogeneity; and I^2^ > 50%, strong heterogeneity) [29]. If heterogeneity was found, the random-effects model was applied. Otherwise, the fixed-effects model was used. In addition, we also investigated potential sources of heterogeneity through meta-regression analysis and subgroup analysis. Sensitivity analyses were performed with the exclusion of studies that had the highest weight, the highest or lowest estimates, the largest sample size, or the studies for which data were acquired through calculation. The Begg’s funnel plot method was used to investigate any possible publication bias. For all analyses, a two-sided *p* value less than 0.05 was considered to be statistically significant. All analyses were performed using STATA version 12.0 software (Stata Corporation, College Station, TX).

## Results

### Literature selection and characteristics

**Figure 1:**
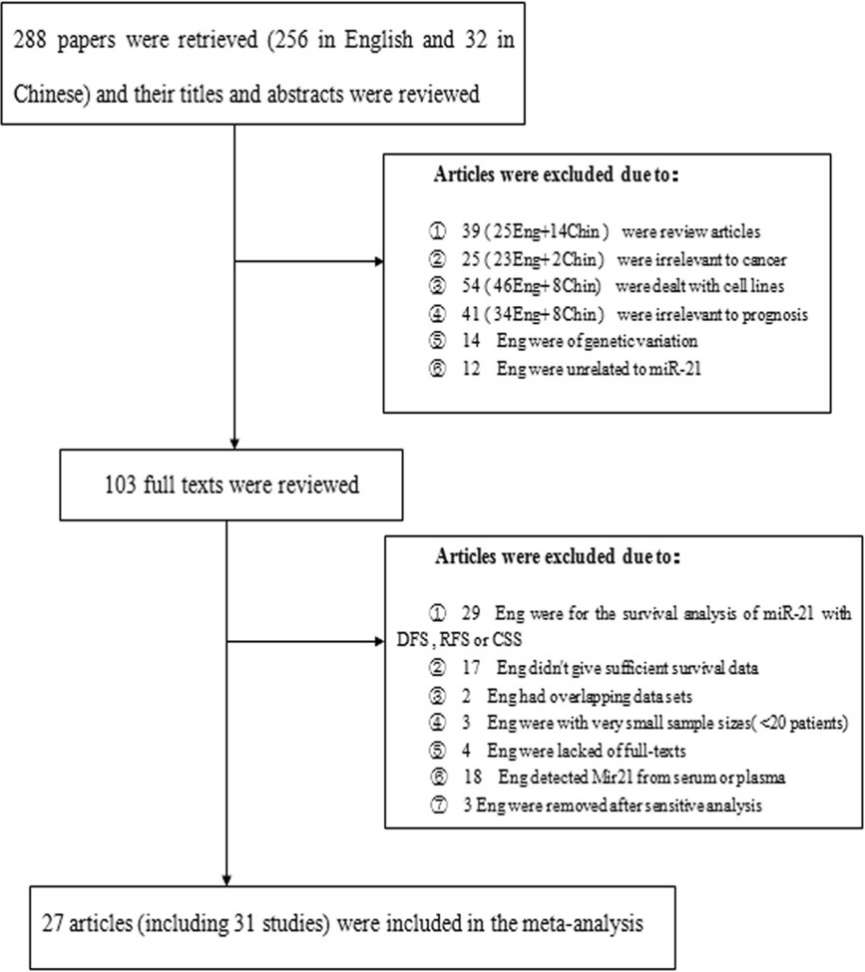
**Flow diagram illustrating the screening and selection process.**

A total of 288 potentially relevant citations, including 256 reports in English and 32 in Chinese, were retrieved after the initial database search using the search strategies described previously. The titles and abstracts of relevant articles were read by two authors independently. A total of 185 citations were excluded from analysis after the first screening based on abstracts or titles (39 were review articles; 25 were irrelevant to cancer; 54 dealt with cell lines or animals; 41 were irrelevant to prognosis; 14 did not study tumour tissues; 12 were unrelated to miR-21), leaving 103 citations for further full text evaluation. Upon further review, 73 articles were eliminated (29 described survival analysis of miR-21 with DFS, RFS or CSS; 17 did not give sufficient survival data; 2 had the overlapping data sets; 3 had a very small sample size; 4 lacked full text; 18 detected miR-21 expression the index from serum or plasma). Then after sensitive analysis as follows, three publications were removed. As a result, 27 eligible studies [11, 13, 30–55] including 31 cases were included for the final analysis. The flow chart for the studies is shown in Figure 1.

The basic characteristic descriptions of the 36 eligible studies are summarised in Table 1. Briefly, these studies were conducted in 11 countries (13 cohorts were Asian populations and 18 cohorts were European and American populations, and they were published between 2003 and 2014. Study sample sizes ranged from 25 to 345 patients (median sample size, 105.5 patients). A total of 18 cohorts were of I-IV stage or of all stages. Most studies investigated miR-21 by quantitative reverse-transcription polymerase chain reaction (qRT-PCR). Overall, 21 cohorts reported miR-21 as an indicator of poor prognosis, while the other 10 showed no significant impact of miR-21 on overall survival.

**Table 1:** **Baseline characteristics of the eligible studies evaluating miR-21 expression and OS**

Study & year	Cancer	Cancer type(system)	Sample size	Country	Stage	Age	Follow-up (months)	Comparisons	Cut off value	Method
Nagao 2012 [17]	PDAC	Other	79	Japan	I-IV	65	40	NG	Mean	qRT-PCR
Shibuya 2010 [31]	CRC	Digestive	156	Japan	^*^A-D	65	60	Normal tissues&	Mean	qRT-PCR
Gao 2010 [11]	NSCLC	Respiratory	47	China	I-III	64	60	Normal tissues	Median	qRT-PCR
Childs 2009 [32]	HNSCC	Other	104	USA	I-IV	60	60	Normal tissues	Mean	qRT-PCR
Yan 2008 [33]	BC	Breast	113	China	I-III	48	66.2	Normal tissues	Mean	qRT-PCR
Lee 2011 [35]	BC	Breast	109	Korea	I-III	48	100	NG	Mean	qRT-PCR
Jiang 2011 [36]	Melanoma	Other	106	China	I-IV	60	60	NG	Median	qRT-PCR
Zhi 2010 [37]	Astrocytoma	Other	124	China	I-IV	47.8	35.2	NG	Median	qRT-PCR
Jamieson 2012 [38]	PDAC	Other	58	UK	II,III	65	23.9	Normal tissues	Median	qRT-PCR
Schetter 2008 [39]	CRC	Digestive	84	USA	I-IV	64.6	68.0	Normal tissues	Highest tertile	qRT-PCR
Schetter 2008 [39]	CRC	Digestive	113	HK	I-IV	55.8	84.6	Normal tissues	Dichotomize	Microarray
Faltejskova 2012 [41]	CRC	Digestive	44	Czech	I-IV	67	84	Normal tissues	Median	qRT-PCR
Chen 2013 [42]	CRC	Digestive	195	Taiwan	I-IV	66	60	Normal tissues	Mean	qRT-PCR
Toiyama 2013 [43]	CRC	Digestive	186	Japan	I-IV	67	60	Normal tissues	0.0031	qRT-PCR
Markou 2008 [13]	NSCLC	Respiratory	48	USA	I-IV	60	50	Healthy controls	2-fold	qRT-PCR
Liu2012 [44]	NSCLC	Respiratory	70	China	I-IV	60	30	Healthy controls	2-fold	qRT-PCR
Saito2011 [45]	NSCLC	Respiratory	89	USA	I-III	63.6	80	Normal tissues	Median	qRT-PCR
Saito2011 [45]	NSCLC	Respiratory	37	Norway	I-III	64.4	80	Normal tissues	Median	qRT-PCR
Saito2011 [45]	NSCLC	Respiratory	191	Japan	I-III	59.6	80	Normal tissues	Median	qRT-PCR
Markou 2013 [46]	NSCLC	Respiratory	48	Greece	I-IV	60	40	Normal tissues	6.3-fold	qRT-PCR
Wu2013 [56]	Glioma	Other	152	China	I-IV	45.1	60	Normal tissues	Mean	qRT-PCR
Papaconstantinou2012 [57]	PC	Other	88	Greece	I-IV	66.5	40	Normal tissues	Mean	qRT-PCR
Karakatsanis2011 [48]	HCC	Digestive	60	Greece	I-IV	60	50	Healthy controls	Mean	qRT-PCR
Kadera2013 [49]	PDAC	Other	153	USA	I, II,IV	65	42	Healthy controls	Median	ISH
Markou 2014 [58]	BC	Breast	112	Greece		60	75	Normal tissues	Median	qRT-PCR
Akagi2013 [50]	LC	Respiratory	67	USA	I	64.9	60	Normal tissues	Median	qRT-PCR
Akagi2013 [50]	LC	Respiratory	25	Norway	I	64.0	60	Normal tissues	Median	qRT-PCR
Bovell2013 [51]	CC	Digestive	345	USA	IV	65	17	Normal tissues	Mean	qRT-PCR
Capodanno 2013 [52]	NSCLC	Respiratory	80	Italy	I-IV	67	32	Normal tissues	Median	qRT-PCR
Faragalla2012 [53]	RCC	Digestive	121	Canada	I-III	62.4	52.8	Normal tissues	40th percentile	qRT-PCR
Mathe 2009 [55]	ESCC	Digestive	69	Two countries	I-IV	62	62.5	Normal tissues	Dichotomize	qRT-PCR

*Abbreviations*: PDAC pancreatic ductal adenocarcinoma, CRC colorectal cancer, NSCLC non-small cell lung cancer, HNSCC head and neck squamous cell carcinoma, CC colon cancer, RC rectal cancer, HCC hepatocellular carcinoma, ESCC oesophageal squamous cell carcinoma, BC breast cancer, PC pancreatic cancer, RCC renal cell carcinoma, ^*^Duke’s stage, ISH In Situ Hybridization, NG Not given, &Adjacent noncancerous tissues were procured from patients.

### Quality assessment and meta-analysis

**Figure 2:**
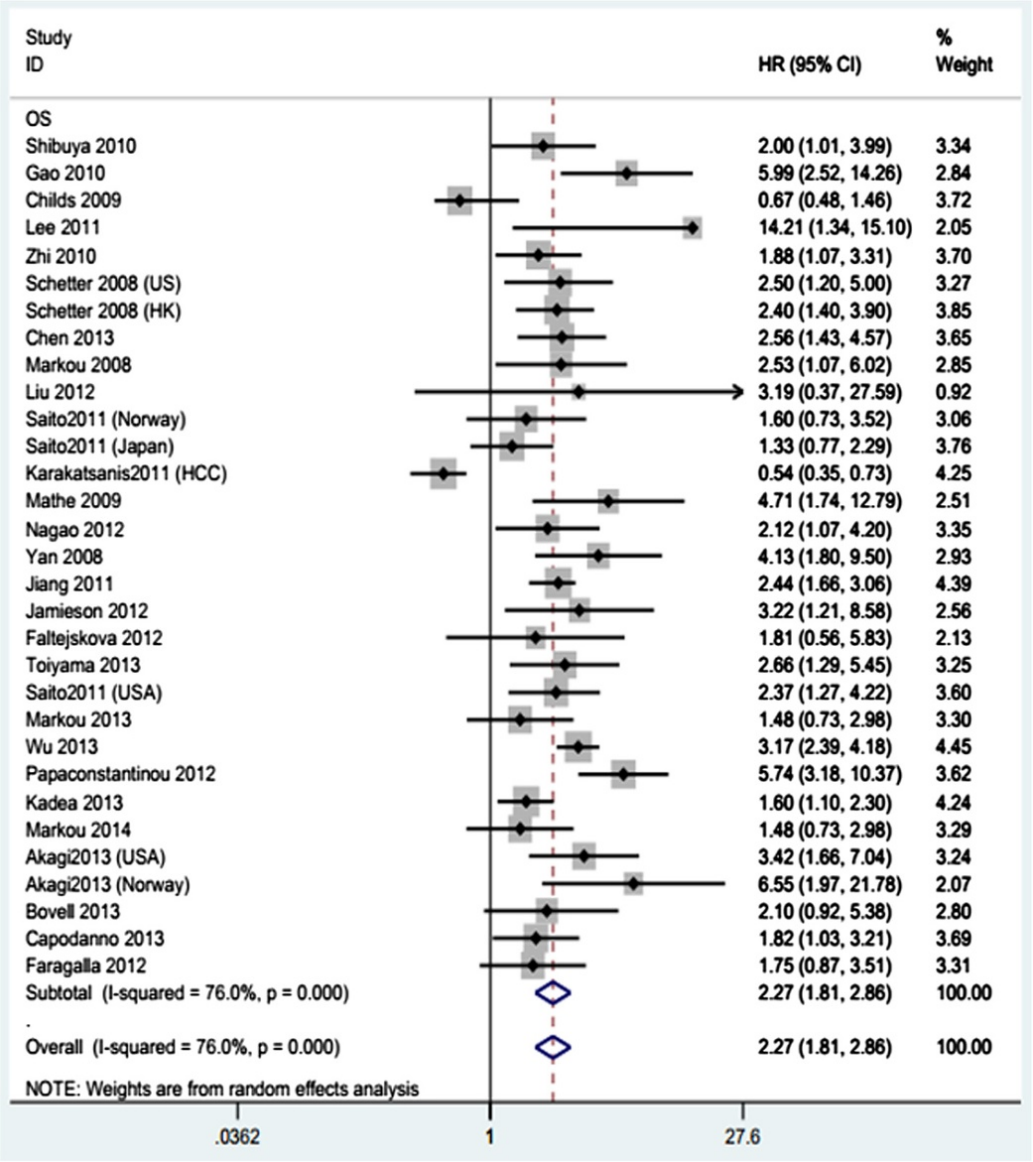
**Meta-analysis of the association between miR-21 expression and prognosis.** Meta-analysis of the forest plot showing the association between miR-21 and overall cancer survival. The squares represent the size of the study and are centred on the HR. The whiskers represent the 95% CIs. A random effects (RE) model was used, and the x-axis shows the Hazard ratio.

REMARK was used a guideline rather than a scoring scale, so the assessment was a qualitative process rather than a quantitative one (Additional file 2: Table S2). Instead of grading every published report and ranking their quality as "high" or "low", we carried out an assessment emphasising the analysis and presentation of the studies to prevent the inclusion of inferior data which would influence the accuracy of the meta-analysis. Two studies were eliminated during this procedure due to their small sample size and poor quality of data [12, 59]. In addition, when using the random-effects model due to the significant heterogeneity of the studies, dismal survival outcomes were observed for tumour patients with miR-21 overexpression. The pooled HRs and CIs were 2.27(1.81-2.86), with I^2^ values of 76.0%, and Figure 2 shows the results of the forest plot explained above.

### Assessment of heterogeneity and subgroup analysis

Highly significant heterogeneity was detected when all studies were pooled (I^2^ = 76.0%), signifying that the variation was due to heterogeneity rather than chance. To make a conservative estimate, a random-effect model rather than a fixed-effect model was used to account for the highly significant inter-study heterogeneity to summarise the prognostic value of miR-21 across studies. When all study populations were combined, dismal survival outcomes were observed with the overexpression of miR-21 (Figure 2). There was evidence of significant inter-study heterogeneity (*p* = 0.001, I^2^ = 76.0%).

Considering the substantial heterogeneity exhibited in the trials aggregated with respect to the overall survival, meta-regression and subgroup analyses were conducted to explore the heterogeneity of the covariates including the publication year, study location, number of patients, mean age, follow-up, cut-off value of miR-21, cancer category and stage (Table 2). Ultimately, the study age might be a source of heterogeneity (Adj R^2^ = 10.63%). The results showed that combined HR of the Asian population was 2.27(1.81,2.86) with I^2^ = 76.0%. We also tried to use other grouping terms to explore the prognostic role of miR-21, such as TNM stage, publication year, CEA (cut-off value) *et al*. However, no clinical significance could be found.

**Table 2:** **Meta-regression and subgroup analysis of the studies reporting the association between microRNA-21 and the overall survival of cancer patients**

Stratified study	No. of studies	Pooled HR(95% CI)		Meta-regression			Heterogeneity	
		Fixed-Model	Random-Model	Tau ^2^	Adj R ^2^ (%)	***p***-value	I ^2^ (%)	***p***-value
**Year**				0.246	3.79	0.350		
>2012	17	2.48(2.17,2.83)	2.46(2.02,2.99)				43.0	0.031
≤2012	14	1.53(1.29,1.81)	2.13(1.36,3.31)				83.3	0.001
**CEA (cut-off value)**				0.268	−4.75	0.848		
Mean & Median	25	2.06(1.84,2.30)	2.31(1.77,3.03)				80.4	0.001
Others	6	2.11(1.53,2.92)	2.11(1.53,2.92)				0.0	0.822
**Sample size**				0.268	−4.48	0.216		
>100	15	1.85(1.53,2.22)	2.53(1.59,4.02)				82.1	0.001
<=100	16	2.18(1.92,2.48)	2.12(1.67,2.69)				66.4	0.001
**Race**				0.260	−1.68	0.537		
Yellow	12	2.63(2.26,3.07)	2.67(2.07,3.42)				50.9	0.021
White	18	1.63(1.41,1.88)	1.94(1.40,2.71)				78.7	0.001
**Age**				0.229	10.63	0.391		
<=60	12	2.42(2.08,2.82)	2.52(2.08,3.06)				32.2	0.088
>60	19	1.80(1.56,2.07)	1.90(1.21,3.01)				87.9	0.001
**TNM stage**				0.263	−2.78	0.362		
I-IV	17	1.98(1.76,2.23)	2.16(1.16,2.92)				80.3	0.001
Others	14	1.22(1.15,1.30)	1.86(1.53,2.26)				80.7	0.001
**Cancer category**				0.258	−0.55	0.421		
Digestive system	13	2.35(1.96,2.81)	2.43(1.95,3.03)				28.1	0.162
Respiratory system	11	1.82(1.47,2.26)	2.08(1.40,3.08)				66.9	0.001
Breast cancer	3	3.07(1.88,5.01)	4.08(1.23,13.48)				81.4	0.005
Others systems	4	1.89(1.60,2.25)	1.68(0.76,3.73)				95.0	0.001
**Detect Method**				0.270	−5.34	0.686		
qPCR	29	2.27(1.81,2.86)	2.07(1.86,2.29)				76.0	0.001
others	2	------	------	------	------	------		
**Overall**	31	2.07(1.86,2.29)	2.27(1.81, 2.86)				76.0	0.001

*Abbreviations*: CI confidence interval, HR hazard ratio.

### Sensitivity analysis and publication bias

**Figure 3:**
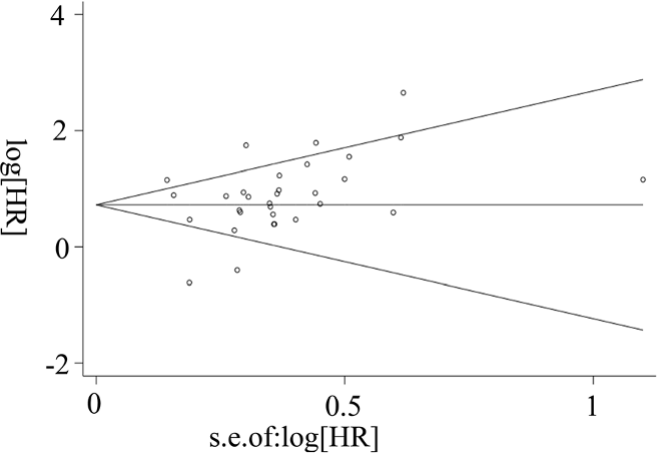
**Begg’s funnel plot of publication bias.** Funnel plot for the visual assessment of the presence of publication bias associated with all of the studies included in the meta-analysis. The funnel graph plots the log of the hazard ratio (HR) against the standard error of the log of the HR (an indicator of the sample size). The open circles indicate the individual studies. The line in the centre represents the pooled HR. Egger’s test for publication bias was not significant (p = 0.174).

The Begg's funnel plot method was applied to detect publication bias in the meta-analysis. No bias was found in any of the included studies (*p* =0.174) (Figure 3). In addition, sensitivity analysis was also conducted (Additional file 3: Figure S1), and we found that when three studies with four cases [34, 40, 54] were discarded, the outcome of the sensitivity analysis was more stable.

## Discussion

Accurate prognostic factors and their predictive functions are particularly valuable in patients with some specific types of cancer which have widely varying outcomes and for which systemic adjuvant therapy might be important. The differentiation of high-risk patients from low-risk patients may help us make a sensible decision to balance treatment with further adjuvant therapy and the toxic side-effects inflicted on patients [60]. MiR-21 is an exciting potential new biomarker of prognosis in malignancies, and molecular studies have been encouraging. While some studies found that miR-21 was significantly associated with patient survival, other studies did not find any significant results for miR-21.

Although a similar meta-analysis on the prognostic value of miR-21 in various types of cancer was reported three years ago [61], there were several problems with the analysis that adversely impacted its quality. First, this study did not describe the heterogeneity among the eligible studies, while the between-study heterogeneity would have a profound influence on the validity of the conclusion. Second, the up-regulation of miR-21 was found in both tumour tissues and non-tumour tissues such as plasma and serum; however, in the absence of a proven correlation between these two sources of tissues, it is not rational to combine their results together without any explanation or discussion [62]. Third, one eligible study emphasised the interaction and combined effect of miR-21 and other factors instead of the independent role of miR-21 in prognosis [63]. In addition, numerous studies on the association between miR-21 and prognosis have emerged since this meta-analysis was published. As described above, carrying out a new systematic review and meta-analysis on this issue was deemed essential. We were able to conduct our meta-analysis on a larger sample size and with a more appropriate method to accurately evaluate the role of miR-21 in the prognosis of cancer.

When we stratified the studies according to the different possible contributors through meta-regression and subgroup analysis, none of the studies had a definitive explanation for the heterogeneity. Generally, the high degree of heterogeneity was probably due to the difference in the baseline characteristics of the included patients (age, histological type, differentiation or tumour stage, race or location, the sample size, the detection methods and the duration of follow-up). In addition, the lack of detailed information about baseline characteristics as a result of non-standardised reporting likely contributed to diversity across the studies as well. Moreover, studies that do not extend their analysis beyond univariate survival analysis are therefore less valuable because these confounders between miR-21 and OS did not distribute equally in each group for the variations in author's clinical experience, adjustment method and the innate characteristics of different cancers. These issues contribute to inaccuracies associated with HR estimation, and therefore, the pooling of results may produce bias and heterogeneity. As a result, the diversification of adjusted factors across studies along with a statistical adjustment for the different clinicopathologic factors included in multivariate analysis might have contributed to the significant heterogeneity.

In addition, traditional survival analysis techniques (Kaplan-Meier, log-rank test) rely on variable dichotomisation into high or low values or splitting variables into multiple bins. In addition, cut-off point selection itself could potentially influence the prognostic value of the proposed association [47]. Due to the lack of a clear and widely agreed upon cut-off definition, the researchers preferred to arbitrarily create one themselves in the laboratory, so the cut-off point and the accuracy of the value varied between studies [24]. All of the above cases made the interpretation difficult because patients with the same values would be considered to have high miR-21 expression in some studies but low expression in others [64]. In essence, estimates derived from different tumour analyses are not comparable, not to mention that they should not be combined in a meta-analysis. Therefore, the assessment of miR-21 expression must be considered to be a potential source of heterogeneity. Thus, adopting a consensus cut-off value for miR-21 expression could facilitate the replication of results. For miR-21 to be a useful predictive biomarker in clinical practice, a single clearly defined protocol needs to be developed and validated to allow the comparison of outcomes across studies.

Although the Begg’s test suggested a *p* value of 0.174, the funnel plot provides some slight evidence of asymmetry between the included studies, which indicates that some epidemiological research bias exists. We attempted to minimise publication bias by performing the literature search as completely as possible using PubMed and Chinese Biomedicine databases, without language or date of publication restrictions. However, limitations still existed as the total number of included studies and the total sample size were relatively small. In addition, we did not extend the search to unpublished data that would likely include increased proportions of null results. Positive results tend to be more acceptable by journals, whereas negative results are often rejected or are not even submitted for review. As already highlighted, the negative studies reported less detailed results, making them unlikely to be evaluated. What's more, the language of publication also introduced bias because positive results tend to be published in English-language journals. Although our search was conducted without language restriction, all of the studies included in the meta-analysis were published in English. As is known to all, the line across the top of a funnel should be representative of the pooled effect.

Our study identified several limitations that must be addressed. First, inadequate sample size was a frequent problem in the studies included in our analyses, with only 15 of the 31 studies reporting outcomes from over 100 patients. While pooling data may in part address deficiencies in individual study sample size, smaller studies are more likely to generate heterogeneity, as we observed. Second, our study used data from published studies rather than individual patient data (IPD), which limited our ability to explore the potential for confounding by various demographic and clinical factors (e.g., ethnicity, disease stage, differentiation and treatment regimes). By contrast, IPD based meta-analysis can be used to analyse all of the data in a consistent manner and includes data from unpublished studies. A careful collaborative reanalysis of the raw data from several good studies may be more valuable than a more superficial review that mixes good and poor studies [64]. Additionally, this study was predominantly based on the findings of observational studies. In particular, a considerable portion of the included studies were retrospectively accrued cohorts, which inherently contained greater potential for confounding than do randomised controlled trials. This issue led to conflicting results and also influenced the authentic prognostic value of miR-21 alterations, providing a lower level of evidence than desired. In addition, four publications had a slightly shorter follow-up time with a duration less than three years. However, we found that in the Capodanno study (2013) and Bowell study (2013), the majority of patients were diagnosed with locally advanced disease. Pancreatic ductal adenocarcinoma (PDAC, Jamieson [38]) and astrocytoma (Zhi [37]), of which grade III and IV constitute 62% of cases, are two of the most aggressive malignancies. The prognosis of these patients is quite poor. Finally, quality assessment tools for examining prognostic and predictive biomarker studies do not currently exist [65], and published articles have often lacked sufficient information to allow an adequate assessment of the quality of the study or the generalisation of the study results. The quality of pooled studies significantly influences the level of confidence of meta-analyses. Therefore, we performed a methodological assessment of the studies to avoid including some poor quality studies in general instead of scoring each individual study [65] according to the REMARK method [25, 27] and the explanation and elaboration that were recently published [66].

## Conclusion

MiR-21 overexpression was found to be associated with decreased overall survival in patients with a wide variety of tumour categories in the present systematic review with meta-analysis. As this research is limited to patients who received surgical treatment without any adjuvant therapy, the miR-21 expression status is a direct outcome of cancer itself and reflected the impact of miR-21 on cancer progression. Therefore, MiR-21 expression is a potentially useful biomarker for predicting prognosis and is a promising prognostic tool to help clinicians make difficult therapeutic decisions. In addition, although we excluded studies that did not include sufficient survival data, we noted that some of them contain negative conclusions about the prognostic role of miR-21. Therefore, large adequately designed prospective studies, both observational cohorts and clinical trials that employ standard methodology, are now urgently needed to substantiate our conclusions. The exact role of miR-21 expression needs to be determined by an appropriate multivariate analysis taking into account the classical well-defined prognostic factors for each type of cancer. Additionally, international consensus is urgently required to mandate a homogeneous miR-21 assessment methodology, to enable comparisons and the combination of large, prospectively planned individual patient data meta-analyses. These types of studies may help determine if miR-21 expression might be more appropriate and better used in clinical decision-making for tumour patients.

## Electronic supplementary material

Additional file 1: Table S1: Data extraction from the eligible studies. (DOCX 41 KB)

Additional file 2: Table S2: The PRISMA Guideline for reporting this systematic review and meta-analysis. (DOCX 26 KB)

Additional file 3: Figure S1: Sensitivity analysis of all of the studies. (TIFF 5 MB)

## Abbreviations

MiR-21: microRNA-21
OS: Overall survival
HR: Hazard ratio
95% CI: 95% confidence interval
miRNAs: MicroRNAs
PDCD4: Programmed cell death 4
TPM1: Tropomyosin1
PTEN: Phosphatase and tensin homolog
MOOSE: Meta-analysis of Observational Studies in Epidemiology
qRT-PCR: Quantitative reverse-transcription polymerase chain reaction
CEA: Cut-off value
IPD: Individual patient data
PDAC: Pancreatic ductal adenocarcinoma
CRC: Colorectal cancer
NSCLC: Non-small cell lung cancer
HNSCC: Head and neck squamous cell carcinoma
CC: Colon cancer
RC: Rectal cancer
HCC: Hepatocellular carcinoma
ESCC: Oesophageal squamous cell carcinoma
BC: Breast cancer
RCC: Renal cell carcinoma
*: Duke’s stage
ISH: In Situ Hybridisation
NG: Not given
&: Adjacent noncancerous tissues were procured from patients.
