# Supplementary material for: MicroRNA-21 and the clinical outcomes of various carcinomas: a systematic review and meta-analysis
Source: BMC Cancer. 2014 Nov 7;14:819. doi: 10.1186/1471-2407-14-819 (PMC4232634; doi:10.1186/1471-2407-14-819)
Supplement: Supplementary file 1 — Additional file 1: Table S1: Data extraction from the eligible studies. (DOCX 41 KB) [file 12885_2014_4987_MOESM1_ESM.docx]

**Table S1. Data extraction form of eligible studies.**

| **Part I The information of literatures** | | | |
| --- | --- | --- | --- |
| **Tilte/Number** |  | | |
| **First Author** |  | | |
| **Country** |  | | |
| **The Journal** |  | | |
| **Pub Year** |  | | |
| **Part II The baseline information of patients** | | | |
| **Tumor** |  | | |
| **Design** |  | | |
| **Sample** |  | **Location** |  |
| **Sex** |  | | |
| **Stage** |  | | |
| **Median age** |  | | |
| **Follow-up** |  | | |
| **Treatment** |  | | |
| **The test** |  | | |
| **Part III The data from survival analysis** | | | |
| **HR** | **（adjusted or unadjusted or both）** | | |
| **95%CI** |  | | |
| **Adjusted factor** |  | | |
| **P value** |  | | |
| **High ：Low** |  | | |
| **supplement** |  | | |

227 papers were retrieved (196 in English and 31 in Chinese) and their titles and abstracts were reviewed

Articles were excluded due to：

1. 32 ( 19Eng+13Chn ) were review articles
2. 19 ( 17Eng+ 2Chn ) were irrelevant to cancer
3. 51 ( 43Eng+ 8Chn) were dealt with cell lines
4. 41 ( 34Eng+ 7Chn ) were irrelevant to prognosis
5. 17 Eng were not tumor tissues
6. 6 Eng were dealt with animals

61 full texts were reviewed

Articles were excluded due to：

1. 20Eng lacked the survival analysis of miR-21 with OS
2. 10Eng didn't apply an average value as the cut-off
3. 6Eng lacked information to estimate HR and 95%CI
4. 2Eng didn't focused on its independent role
5. 2Eng were with a very small sample size <30 patients
6. 1Chn had the same cohort with 1 Eng

20 studies were included in a meta-analysis

**Figure 1. Flow chart of literature selection to identify studies eligible for pooling.**

| **Table 1.Baseline characteristics of eligible studies evaluating miR-21 expression and OS** | | | | | | |  |
| --- | --- | --- | --- | --- | --- | --- | --- |
| **Study** | **Cancer** | **Sample size** | **Location** | **Stage** | **Age** | **Follow-up (month)** | **Cutoff value** |
| Nagao 2012 | PDAC | 65 | Japan | I-IV | 65(40-80) | 40 | mean |
| Shibuya 2010 | CRC | 156 | Japan | Dukes'A-D | 65(25-68) | 44(2-84) | mean |
| Gao 2010 | NSCLC | 47 | China | I-III | — | 30-65 | median |
| Childs 2009 | HNSCC | 104 | USA | I-IV | — | 60 | mean |
| Voortman 2010 | NSCLC | 631 | 14 coutries | I-III | — | 96 | median |
| Mathe(SCC) 2009 | Esophageal cancer | 69 | USA，Japan | I-IV | — | — | median |
| Mathe(ADC) 2009 | Esophageal cancer | 63 | USA，Canada | — | — | — | median |
| Rossi 2010 | CLL | 104 | USA | Rai 1-4 | 62(37-89) | 20(0-88) | median |
| Rossi(validation) 2010 | CLL | 80 | USA | Rai 1-4 | — | — | median |
| Yan 2008 | Breast cancer | 113 | China | I-III | 48(29-74) | 66.2(10.4-81.0) | mean |
| Giovannetti 2010 | PDAC | 59 | Netherlands | I-IV | 63(32-83） | 17.3(1.6-60.5) | median |
| Li 2009 | TSCC | 103 | China | I-IV | — | 70 | mean |
| Gao 2011 | NSCLC | 30 | China | I-III | 63 | 60 | median |
| Valladares-Ayerbes 2011 | Gastrointestinal | 32 | Spain | I-IV | 62.5(45-76） | 38(0.5-97) | mean |
| Lee 2011 | Breast cancer | 109 | Korea | I-III | 48 | 100 | mean |
| Jiang 2011 | Gastric cancer | 55 | China | III,IV | 62.6 | — | mean |
| Jiang 2011 | Cutaneous malignant melanoma | 86 | China | I-IV | — | 60 | median |
| Zhi 2010 | Astrocytoma | 124 | China | I-IV | 47.8 | 35.2(1-98) | median |
| Jamieson 2012 | PDAC | 48 | UK | II,III | — | 23.9 | median |
| Tomimaru 2010 | HCC | 30 | Japan | advanced | 56.6 | 18.2 | median |
| Hamano 2011 | Esophageal cancer | 98 | Japan | I-IV | 61.6 | 28.8(2.3-96.7) | median |
| Lee 2011 | SCLC | 31 | Netherlands | — | 63(38-78) | — | median |
| PDAC, pancreatic ductal adenocarcinoma; CRC, colorectal cancer; NSCLC, non small cell lung cancer; HNSCC, head and neck squamous cell carcinoma; CLL, chronic lymphocytic leukemia; TSCC, tongue squamous cell carcinomas; HCC, hepatocellular carcinoma; SCLC, squamous cell lung carcinoma. | | | | | | | |

| **Table 2. Results of survival analyses by individual study** | | | | |
| --- | --- | --- | --- | --- |
|  | | | | |
| **Study** | **Univariate Analysis** | | **Multivariate Analysis** | |
|  | **HR** | **95% CI** | **HR** | **95% CI** |
| Nagao 2012 | 2.51^*^ | 1.30-4.88^*^ | 2.12 | 1.07-4.20 |
| Shibuya 2010 | 2.99^*^ | 1.62-5.41^*^ | 1.95^*^ | 1.05-3.57^*^ |
| Gao 2010 | 2.71 | 1.39-5.28 | 5.99 | 2.52-14.26 |
| Childs 2009 | 0.67 | 0.48-1.46^#^ | — | — |
| Voortman 2010 | 0.81 | 0.65-1.01 | — | — |
| Mathe(SCC) 2009 | 1.17 | 0.53-2.57 | — | — |
| Mathe(ADC) 2009 | 0.79 | 0.39-1.60 | — | — |
| Rossi 2010 | 2.28 | 1.05-4.97 | 3.47 | 1.35-8.94 |
| Rossi(validation) 2010 | 6.72 | 1.48-30.44 | — | — |
| Yan 2008 | 5.48 | 2.42-12.40 | 4.13 | 1.80-9.50 |
| Giovannetti 2010 | 2.30 | 1.30-4.10 | 3.10 | 1.20-5.30 |
| Li 2009 | — | — | 1.03 | 1.02-1.04 |
| Gao 2011 | 1.25 | 1.09-1.42 | 1.29 | 1.12-1.49 |
| Valladares-Ayerbes 2011 | 1.00 | 0.98-1.02 | — | — |
| Lee 2011 | 5.32 | 0.97-29.03 | 14.21 | 1.34-15.10 |
| Jiang 2011 | 5.88^*^ | 2.22-16.67^*^ |  |  |
| Jiang 2011 | — | — | 2.44 | 1.66-3.06 |
| Zhi 2010 | 1.84 | 1.05-3.22 | 1.88 | 1.07-3.31 |
| Jamieson 2012 | — | — | 3.22 | 1.21-8.58 |
| Tomimaru 2010 | 2.90^$^ | 1.14-7.41^$^ | — | — |
| Hamano 2011 | 1.77^$^ | 1.02-3.15^$^ | — | — |
| Lee 2011 | 0.85^$^ | 0.41-1.76^$^ | — | — |
| HR and associated 95% CI were given as quoted unless stated otherwise, (-) indicated not assessed;  ^*^ estimated result from the reciprocal of data presented in paper;  ^#^ calculated with HR and the P value;  ^$^ obtained from the authors;  HR, hazard ratio; CI, confidence interval. | | | | |

| **Table 3. Meta-analysis and subgroup analysis results** | | | | | | | | | | | | | | | | | | | | | | |
| --- | --- | --- | --- | --- | --- | --- | --- | --- | --- | --- | --- | --- | --- | --- | --- | --- | --- | --- | --- | --- | --- | --- |
|  | | | | | | | | | | | | | | | | | | | | | | |
|  | Meta-analysis of unadjusted HR | | | | | | | | | | | | Meta-analysis of adjusted HR | | | | | | | | | |
| Studies | N cohorts | | | Sample size | Pooled HR | | | 95%CI | | I2(%) | | | N cohorts | | Sample size | Pooled HR | | 95%CI | | I2(%) | | |
| Total | 19 | | | 2000 | 1.63 | | | 1.32-2.01 | | 83.5 | | | 12 | | 1044 | 2.37 | | 1.75-3.23 | | 90.7 | | |
| **Subgroup** | | | | | | | | | | | | | | | | | | | | | | |
| **Location** | | | | | | | | | | | | | | | | | | | | | | |
| Asian | 10 | | | 827 | 2.57 | | | 1.74-3.80 | | 77.1 | | | 9 | | 833 | 2.18 | | 1.57-3.02 | | 91.8 | | |
| Caucasian | 7 | | | 473 | 1.24 | | | 0.84-1.82 | | 71.6 | | | 3 | | 211 | 3.23 | | 1.96-5.34 | | 0.0 | | |
| **Cut-off** | | | | | | | | | | | | | | | | | | | | | | |
| mean | | 7 | | 634 | 2.32 | | | 1.23-4.37 | | 88.9 | | | 5 | | 546 | 2.64 | | 1.23-5.68 | | 89.3 | | |
| median | | 12 | | 1366 | 1.51 | | | 1.15-1.98 | | 72.8 | | | 7 | | 498 | 2.50 | | 1.63-3.84 | | 81.7 | | |
| **Sample size** | | | | | | | | | | | | | | | | | | | | | | |
| small(<80) | | 11 | | 561 | 1.58 | | | 1.22-2.04 | | 82.7 | | | 5 | | 249 | 2.57 | | 1.39-4.76 | | 80.6 | | |
| large(≧80) | | 8 | | 1439 | 1.86 | | | 1.09-3.16 | | 85.4 | | | 7 | | 795 | 2.57 | | 1.47-4.50 | | 91.9 | | |
| **Stage** | | | | | | | | | | | | | | | | | | | | | | |
| I-IV | | 10 | | 891 | 1.73 | | | 1.19-2.50 | | 81.6 | | | 7 | | 697 | 2.04 | | 1.27-3.27 | | 89.7 | | |
| I-III | | 5 | | 930 | 1.81 | | | 1.10-2.97 | | 88.4 | | | 4 | | 299 | 4.24 | | 1.36-13.19 | | 90.8 | | |
| Advanced | | 2 | | 85 | 4.02 | | | 2.02-8.03 | | 1.4 | | | — | | — | — | | — | | — | | |
| **Cancer** | | | | | | | | | | | | | | | | | | | | | | |
| LGI | | 6 | | 397 | 2.42 | | 1.28-4.56 | | | | 88.9 | | 4 | | 328 | 2.38 | | 1.66-3.42 | | **0.0** | | |
| UGI | | 4 | | 334 | 1.02 | | 0.64-1.65 | | | | 53.7 | | — | |  | — | | — | | — | | |
| Breast | | 2 | | 222 | 5.45 | | 2.61-11.38 | | | | **0.0** | | 2 | | 222 | 7.06 | | 2.31-23.45 | | 63.2 | | |
| Lung | | 4 | | 739 | 1.17 | | 0.79-1.72 | | | | 83.5 | | 2 | | 77 | 2.61 | | 0.58-11.71 | | 91.5 | | |
| CLL | | 2 | | 184 | 3.2 | | 1.20-8.54 | | | | 35.6 | | — | | — | — | | — | | — | | |
| HR, hazard ratio; CI, confidence interval; LGI, lower gastrointestinal; UGI, upper gastrointestinal; CLL, chronic lymphocytic leukemia. | | | | | | | | | | | | | | | | | | | | | | |
| **Table 4. Sensitivity Analysis results** | | | | | | | | | | | | | | | | | | | | | |  |
|  | | | | | | | | | | | | | | | | | | | | | |  |
|  | | | Univariate Analysis | | | | | | | | | | | Multivariate Analysis | | | | | | | |  |
|  | | | Exclusion of studies | | | HR | | | 95%CI | | | I^2^ | | Exclusion of studies | | | HR | | 95%CI | | I^2^ |  |
| Highest weight | | | Valladares-Ayerbes 2011 | | | 1.82 | | | 1.38-2.40 | | | 79.1 | | Li 2009 | | | 2.77 | | 1.92-4.01 | | 80.4 |  |
| Highest HR | | | Rossi(validation) 2010 | | | 1.59 | | | 1.29-1.95 | | | 83.5 | | Lee 2011 | | | 2.16 | | 1.60-2.90 | | 90 |  |
| Lowest HR | | | Childs 2009 | | | 1.73 | | | 1.39-2.15 | | | 84.1 | | Li 2009 | | | 2.77 | | 1.92-4.01 | | 80.4 |  |
| Largest samplesize | | | Voortman 2010 | | | 1.78 | | | 1.41-2.24 | | | 83.8 | | Shibuya 2010 | | | 2.42 | | 1.75-3.34 | | 91.2 |  |
| Calculated data | | | Childs 2009 | | | 1.73 | | | 1.39-2.15 | | | 84.1 | | — | | | — | | — | | — |  |
| HR, hazard ratio; CI, confidence interval. | | | | | | | | | | | | | | | | | | | | | |  |

**Figure 2 Funnel plots of Begg's were used to detect publication bias in unadjusted HR(above) and adjusted HR(below).**

**Figure 3. Funnel plots of Begg's after the exclusion of one particular point in unadjusted HR(above) and adjusted HR(below).**
