# Supplementary figures and images for: MicroRNA-21 and the clinical outcomes of various carcinomas: a systematic review and meta-analysis
Source: BMC Cancer. 2014 Nov 7;14:819. doi: 10.1186/1471-2407-14-819 (PMC4232634; doi:10.1186/1471-2407-14-819)

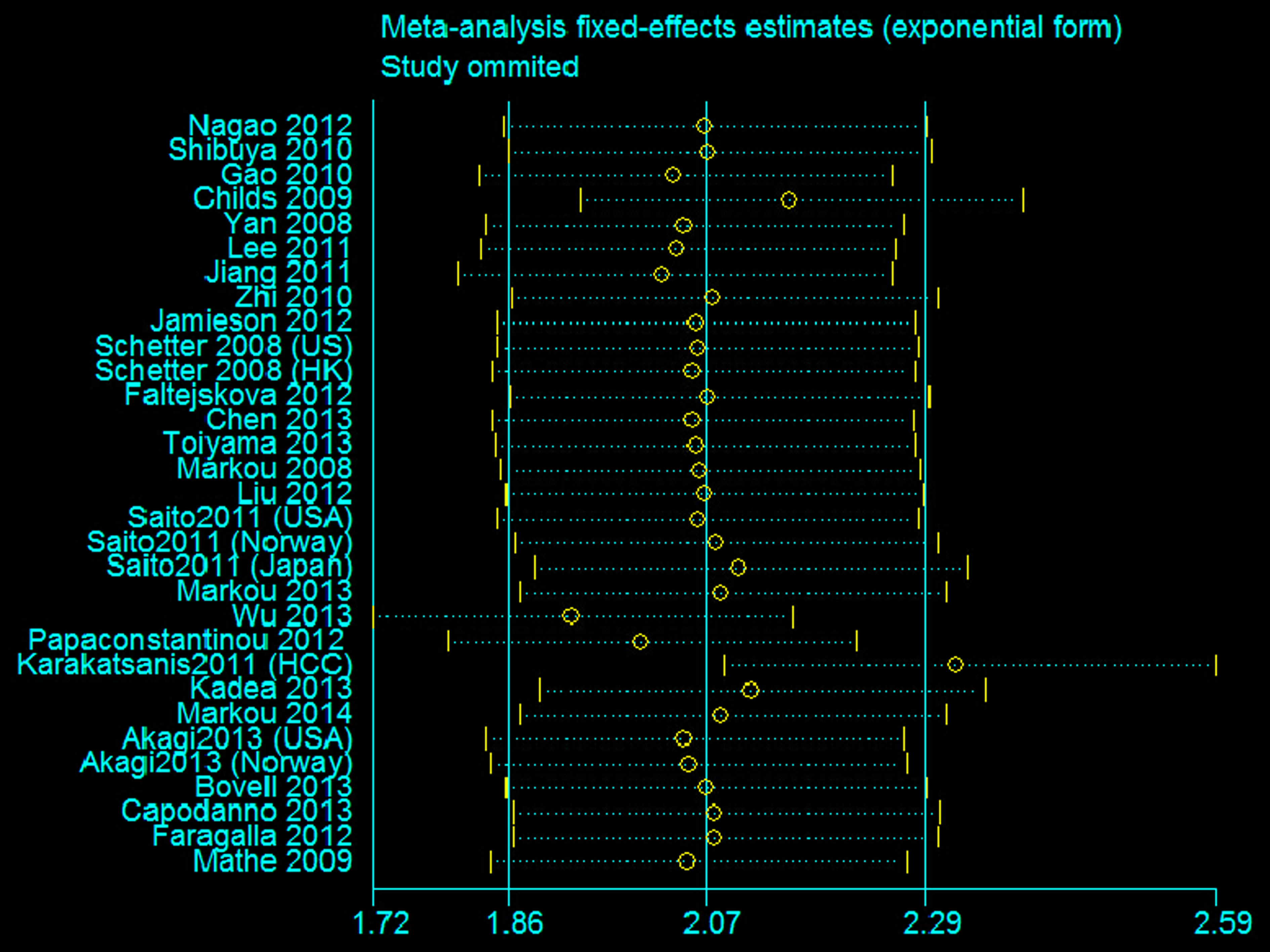

Supplement: Supplementary file 3 — Additional file 3: Figure S1: Sensitivity analysis of all of the studies. (TIFF 5 MB) [file 12885_2014_4987_MOESM3_ESM.tiff]
